# The Impact of Urban Sprawl on Environmental Pollution: Empirical Analysis from Large and Medium-Sized Cities of China

**DOI:** 10.3390/ijerph18168650

**Published:** 2021-08-16

**Authors:** Huan Zhang

**Affiliations:** School of Economics, Nanjing Audit University, No. 86 Yushanxi Road, Jiangpu Street, Pukou District, Nanjing 211815, China; zhanghuan371@163.com

**Keywords:** urban sprawl, environmental pollution, urbanization, Environmental Kuznets Curve, city planning

## Abstract

This research sought to uncover the environmental effects that urban sprawl brings about by using panel data of 35 large and medium-sized cities over the timespan 1998–2019. A nonlinear relationship between urban sprawl and environmental pollution might exist and be shown by constructing the conceptual mathematical model. The panel unit root test indicated that there exists a long-run equilibrium relationship between urban sprawl and environmental pollution. Regression results showed that there is a U-shaped curve relationship between the two core variables for the full sample. The impacts of urban sprawl on environmental conditions have regional heterogeneity. A significant U-shaped curve was discovered for eastern and western regions, while an insignificant inverted U-shaped curve was discovered for the central region. Furthermore, we found a significant U-shaped curve for cities with high and low degree of sprawl. By contrast, we found a significant inverted U-shaped curve for cities with a middle degree of sprawl, which indicated that moderate urban sprawl is beneficial for environmental conditions. We additionally discovered a significant U-shaped curve and inverted U-shaped curve for cities with low and middle pollution, respectively. Fixed-asset investments and foreign direct investments exacerbate urban environmental pollution. Market freedom and industrial structure adjustment are conducive to pollution reduction. The conclusions were consistent when we subdivided the sample by time periods and substituted with a systematic GMM (Generalized Method of Moments) estimation method in the robustness check. Policy recommendations were put forward according to the research findings.

## 1. Introduction

**Figure 1:**
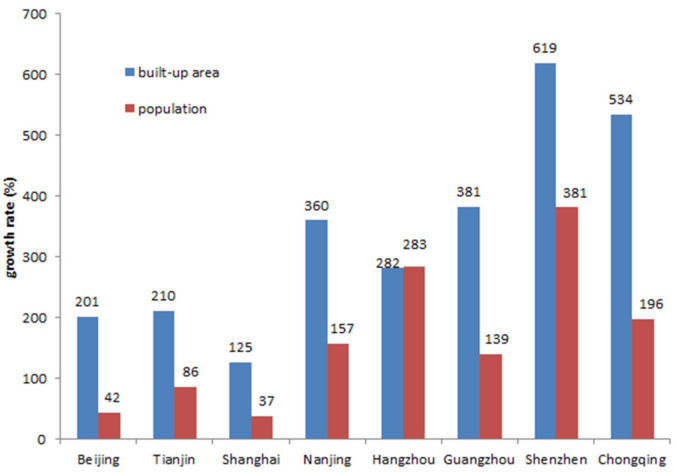
Growth rate of built-up areas and population during 1998–2019 for some large cities in China.

After the economic reform and opening-up policy, with the promotion of China’s rapid urbanization, the level of urbanization and regional development has been greatly improved. The rural society accelerated the transformation to urban society. The number of cities and towns is climbing, and the scale of cities and towns is enlarging. The rapid improvement of urbanization level means that a large amount of the rural population transfer to the city. As a process of population concentration to the city, theoretically speaking, it is conducive to the economical and intensive use of land. However, with the acceleration of China’s urbanization process, the typical fact is the extensive utilization of land. The urban spatial growth in China has a significant feature of non-intensive and disorderly spread. More and more cities have adopted the growth mode of low-density and disorderly outward expansion. Especially in the megacities with urban populations exceeding 2 million and the megacities with an urban population exceeding 1 million, blind spatial expansion is one of the five most serious problems in the process of China’s urbanization. Based on the analysis of the spread and expansion rate of most cities and towns in China, the speed of Land Urbanization “takes the lead” and presents a situation of land being out of control. Except Hangzhou, the growth rate of urban areas in other large and medium-sized cities exceeds that of the population growth rate of the municipal area (Figure 1), which indicates that the phenomenon of low-density spread in large and medium-sized cities is extremely common [1]. In China, a medium-sized city is one whose population varies between 1000,000 and 5000,000, while large cities, including megalopolises, constitute a population of 5000,000 to 10,000,000 and megacities a population over 10,000,000. Large and medium-sized cities have the advantages of a large number of employment opportunities and advanced infrastructure, which constantly attract an influx of population and form industrial agglomeration. However, the expansion of urban construction has not been coordinated in time. A series of negative externalities brought by urban population and industrial agglomeration have seriously affect the quality of life of urban residents.

With the popularization of sprawling spatial growth, urban diseases such as unreasonable land use, traffic congestion, and environmental pollution gradually appear in the process of urban development. In recent years, China has been facing increasingly serious environmental pollution problems while maintaining rapid economic growth. According to the China Environmental Statistics Yearbook 2019, in 2018, China’s total waste water discharge was 73.53 billion tons, the total industrial solid waste discharge was 558,000 tons, the industrial waste gas emission was 68,519 billion square meters, and the sulfur dioxide emission was 18.59 million tons, which marked it as one of the most polluted countries in the world. According to the announcement of 2014 China’s statement of the Environment Bulletin, 161 cities in China have carried out new air quality standard monitoring, but only 16 cities have reached the standard. This shows that China’s “three high” economic development mode of “high growth, high consumption and high pollution” has not been fundamentally changed, which seriously restricts the sustainability of China’s urban development. Therefore, Premier Li Keqiang once put forward, in the 2014 government work report, that like declaring war on poverty, we should firmly declare war on pollution. In the latest government report, Premier Li Keqiang made it clear that China would “resolutely fight the blue sky defense war”.

However, in terms of the current pollution control efforts of China’s cities, there is a wide gap between cities and regions. In high-pollution areas with high coal consumption (such as Shanxi, Hebei, Shandong, and Henan), environmental regulation is relatively low, and thus it is urgent to control environmental pollution. In the past, China’s economic growth mode largely depended on the input of production factors and the consumption of resources. In this mode, environmental protection frequently gives way to regional economic growth. The scarcity of land resources makes the spatial economic attributes of urban land prominent [2]. Under the incentive of GDP (Gross Domestic Product)-oriented performance appraisal, local governments have become highly profit-seeking “entrepreneurial governments”. However, the high land financial revenue source of real estate prosperity has become the focus of development object, and they are keen on the “spread the pie” and “reinforced concrete” urbanization expansion modes. The tentacles of urban expansion began to extend to forests and farmland. Scattered cities continue to consume agricultural land, and the excessive use of energy has created plenty of pollution and destroyed the original urban life. Although the form of urban sprawl is different, its rapid expansion process is reflected in every city in the world, especially in all the cities with high income and developed real estate markets. Therefore, the nexus between urban sprawl and air pollution is a serious and realistic problem faced by local governments. Large and medium-sized cities have high levels of economic development, rapid urbanization processes, rapid development of private transportation, and high frequency and probability of haze pollution, which is representative. This paper aimed to clarify the impact of urban sprawl on air pollution and identify the existence of the Environmental Kuznets Curve (inverted U-shaped curve relationship between per capita income and environmental pollution raised by Simon Kuznets) relationship between them. The remaining sections are organized as follows: Section 2 reviews the past literature works and informs the novelty of the paper; Section 3 proposes the mathematical model; Section 4 introduces materials and estimation methods; Section 5 presents the research results; Section 6 concludes the research findings and puts forward policy recommendations.

## 2. Literature Review

Urban sprawl is an important phenomenon in the process of China’s economic development and urbanization. With the rapid expansion of urban scale, the extensive and inefficient development of construction land increases the bearing pressure of soil and water resources, and the substantial increase of infrastructure construction also aggravates industrial pollution. In addition, the expansion of cities mainly relies on cars for commuting, which leads to more automobile exhaust emissions and more serious air pollution. Since the 1960s, the ecological and environmental problems caused by urban sprawl have attracted extensive attention of scholars, but there are relatively few studies regarding China [3,4]. Generally speaking, urban sprawl has a significant negative impact on environmental quality [5], which mainly destroys the ecological environment by occupying cultivated land, damaging wetlands, and polluting the environment [6], and also leads to serious air pollution such as haze [7]. However, some studies suggest that urban sprawl can also reduce urban environmental pollution by improving energy efficiency [8]. Overall, low-density openness, single-land use, and automobile orientation are the three characteristics of urban sprawl. The existing research on the impact of urban sprawl on environmental pollution mainly includes three aspects: the impact of urban sprawl on energy consumption, the impact of urban sprawl on carbon emissions, and the impact of urban sprawl on air pollution.

### 2.1. Study on the Impact of Urban Sprawl on Energy Consumption

The relationship between urban sprawl and energy consumption is still a controversial topic in urban planning. Some studies [5,9] support the research conclusion of Newman and Kenworthy [10] that, from the perspective of transportation, more compact urban spatial form tends to be more energy-saving. The use of private passenger transport in densely populated cities often results in lower energy consumption and lower greenhouse gas emissions per capita [11]. Song (2005) pointed out that urban sprawl leads to the increase of residential and commercial land. This land use pattern is an important influencing factor of the “urban heat island effect”. At the same time, forest vegetation has a “cooling effect” on the surface temperature of urban environment and surrounding areas. Therefore, he believes that compact urban space can improve energy efficiency and contribute to the sustainable development of cities. On the contrary, McLaren [12] found that urban spatial concentration will also produce negative effects, such as traffic congestion, residential overcrowding, environmental degradation, and other problems, which will increase energy consumption. Similarly, the research of Fallah et al. [13] showed that in the process of rapid urban space expansion, excessively high urban density will lead to “agglomeration diseconomy” such as traffic congestion and high house prices, which will exceed the contribution of agglomeration economy to productivity. Therefore, urban sprawl is conducive to improving total factor productivity.

### 2.2. The Impact of Urban Sprawl on Carbon Emissions

Makido [14] studied the relationship between urban morphology and carbon dioxide in 50 cities in Japan, and found that cities with high income, small population, and high density tend to reduce carbon dioxide emissions. By taking 125 cities with the highest degree of urbanization in the United States as samples, Lee and Lee [15] examined the impact of urban form on residents’ carbon dioxide emissions, and found that urban sprawl was positively correlated with residents’ energy-related carbon dioxide emissions. This is consistent with the conclusion of empirical research on China conducted by Ye et al. [1]. Di Liddo [16] explored the relationship between urban spatial structure and carbon emissions in Italy, and concluded that compact and high-density cities can reduce carbon dioxide emissions from private transportation. Xiao and Zheng [17] explored the relationship between urban construction land expansion and carbon emissions from energy consumption by collecting the data of construction land and energy consumption Wuhan from 2003 to 2013.

### 2.3. Study on the Influence of Urban Sprawl on Air Pollution

According to the existing literature, urban sprawl has aggravated the challenge to the protection of open space, resulting in the deterioration of air quality. Ewing et al. [18] measured the urban sprawl index and studied the impact of urban sprawl on traffic in 83 large cities in the United States from 1990 to 2000. The results showed that low density and vehicle-oriented urban sprawl is not conducive to the formation of good air quality. Burchfield et al. [19] studied the adverse effects of urban sprawl on air pollution in megacities such as Teheran, and found that urban sprawl increased the degree of air pollution. Based on the data of 45 large cities in the United States from 1990 to 2002, Stone [20] proved that the urban air pollutants exceeding the standard in the “big pie” urban sprawl are more serious than those in the compact city. The more compact the city is and the smaller the sprawl degree is, the more likely it is to reduce air pollution emissions. The automobile is the cause of many mechanisms of the “spread the pie” urban sprawl leading to environmental degradation [21]. Using data on PM_2.5_ emissions of 269 cities in China, Yuan et al. [22] examined the relationship between urban form and haze pollution. The results showed that the impact of urban population density on air pollutants depended on city size. Regional transmission exacerbates the air condition if appropriate urban planning is formulated in time. Fan et al. [3] conducted an empirical study on 344 prefecture-level cities in China, and found that urban construction in the northern plain of China should be as compact and continuous as possible, and the multi-center form can effectively reduce air pollution emissions.

### 2.4. Key Findings and Research Contribution

The above literature has laid a foundation for the study of this paper, but there are also some deficiencies. First of all, few studies have put urban sprawl and environmental pollution in the same research framework to investigate the impact mechanism between them. Besides, there is no research result to test the Environmental Kuznets Curve between urban sprawl and environmental pollution. Additionally, for cities with different urban scales, the degree of urban sprawl and environmental pollution vary from high to low. The existing literature does not pay enough attention to the heterogeneity of influencing effects. This is not conducive to the scientific formulation of urban environmental pollution control policies. In comparison, this paper tried to fill the research gap from the following perspectives. To begin with, we put urban sprawl and environmental pollution in the same framework, and theoretically explored the correlation between them. Secondly, we used the panel data of 35 large and medium-sized cities in China from 1998 to 2019, empirically examined the impact intensity of urban sprawl on environmental pollution, and conducted an Environmental Kuznets Curve test on the relationship between urbanization and environmental pollution. Furthermore, we subdivided the sample cities into several subgroups based on the degree of sprawl and environmental pollution to observe the heteroskedastic impacts.

## 3. Model Construction

### 3.1. Urban Sprawl Driven by Government Behavior

It was assumed that the workplace and employment opportunities are concentrated in the city center, and the urban residents are in the peripheral area of the city center, and the commuting distance is from the residence to the core area. According to the theory of welfare economics and the background of China’s fiscal decentralization system, the decision making of local government is based on the pursuit of the maximization of its own interests. Combined with research of Johnson [23], local government maximizes land financial benefits in the process of transforming agricultural land into urban land and land development. Its target profit function is as follows:

(1) $$G=\int_{0}^{T}R(\theta ,t)e^{-\mu t}dt+\int_{T}^{\infty }E(\delta )e^{-\mu t}dt-\int_{0}^{T}r_{\mu t}e^{-\mu t}dt-Ce^{-\mu t}$$

R(θ,t) is the rent level of urban land, $r_{\mu t}$ is the rent cost of non-urban land, μ is the discount rate, T is the time of land development, C is the cost of land development, θ signifies the location, t denotes the time; E(δ) is the external earning, E(δ)≥0, which represents the earning as the increasing function of the economic density of regional population, i.e., ∂E(δ)δ>0. Find the maximum value of the objective function:

(2) ∂G∂T=rμte−μT+μCe−μT−Re−μT−E(δ)e−μT=0

The following can be obtained through sorting and calculation:

(3) $$R(T,\theta ,t)=r_{\mu t}+\mu C-E(\delta )$$

**Figure 2:**
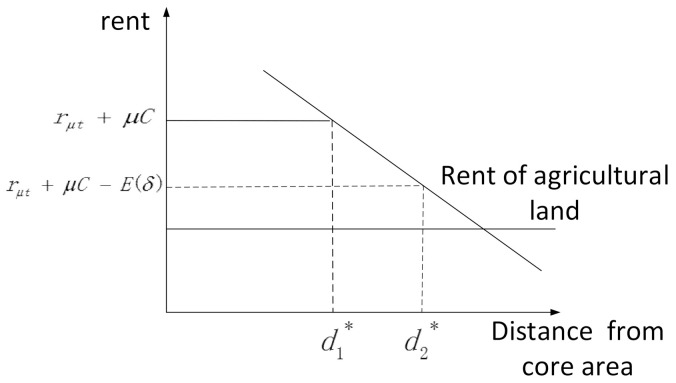
Diagram of urban space expansion.

Under the balanced condition, the rent level of urban land is equal to the agricultural land rent level plus the discounted land development cost minus the external benefits of land development. That is to say, due to the existence of external benefits, the local government’s growth performance is affected by the land’s financial dependence, and there is an impulse to expand the scale of construction land. The urban boundary expands from the original balanced $d^{*}$ to the new balanced $d_{1}{}^{*}$, which makes the expansion speed of urban built-up areas far faster than the population growth, and urban sprawl occurs (Figure 2).

### 3.2. Environmental Effects of Urban Sprawl

This paper expanded the transformation based on the model of Fulton et al. [24] for analysis. Considering from the aspect of environmental pollution, under the influence of an official promotion mechanism, there is a central government and two local governments: the central government has the jurisdiction of promotion rules and the right to set environmental regulations, while the local government needs the economic growth to support financial expenditure; on the other hand, it is also necessary to implement the environmental regulation measures formulated by the central government, so its utility function is affected by land finance, local economic growth, and environmental regulation. The specific settings are as follows:

(4) $$U_{\mu }=\alpha G_{\mu }+\beta Y_{\mu }-R_{\mu }(P-A_{f})$$

Here, U is the total utility obtained by the local government, Y is the regional economic growth, R is the intensity of environmental regulation, P is the environmental pollution, $A_{f}$ is the maximum permitted amount of pollution set by the central government, and $P-A_{f}$ is the negative utility caused by excessive pollution under the environmental protection system.

According to the theory of Environmental Kuznets, there is an “inverted U” relationship between economic growth and environmental pollution, and there is an “inverted U” relationship between land finance and economic growth, which has crossed the turning point [25]; in order to meet the above basic conclusion, the function forms of the two are respectively as follows:

(5) $$P_{\mu }=\eta Y_{\mu }-\rho Y_{\mu }^{2}$$

(6) $$\omega^{2}G_{\mu }=Y_{\mu }^{2}$$

The parameters α, β are the central government’s emphasis on the local government’s land finance and economic growth, and $\alpha_{1}$, $\alpha_{2}\in$(0, 1), and η, ρ, ω are real numbers greater than zero. By combining Equations (4)–(6), we obtain:

(7) $$U=(\alpha +\rho \omega^{2}R)G+RA_{f}+\omega \sqrt{G}(\beta -R\eta )$$

Not hard to find, when R<β/η, dU/dG > 0, indicating that the intensity of environmental regulation is lower than β/η, the government has the intention to expand the financial scale of land. Therefore, only when the intensity of environmental regulation is high can local governments reduce their dependence on land finance. However, from the reality of our country, the intensity of environmental regulation is increasing. Why does the proportion of land finance to local financial revenue remain high? This is closely related to the expectation of the central government for economic growth. Assuming that the central government, on the premise of ensuring economic growth, seeks to minimize the gap between local pollution, its utility function is as follows:

(8) $$U_{MAX}=-\left[\ln(P_{1}+\ln(P_{2})\right]\;\;\;\;\;s.t.\;\;Y_{1}+Y_{2}\geq \xi$$

ξ is the economic growth target set by the central government. By introducing 2.5 and 2.6 into it, and by constructing a Lagrange function, the partial derivatives of $G_{1}$, $G_{2}$ and λ are obtained:

(9) $$U_{MAX}=-\left[\ln(P_{1}+\ln(P_{2})\right]\;\;\;\;\;s.t.\;\;Y_{1}+Y_{2}\geq \xi$$

(10) ηω−2ρω2G12ηωG1−2ρω2G132=λc, ηω−2ρω2G22ηωG2−2ρω2G232=λω, ω(G1+G2)=ξ

The equilibrium value is:

(11) $$G_{1}{}^{*}=G_{2}{}^{*}=\frac{\xi^{2}}{4\omega^{2}}$$

This shows that in the process of the game between local government and central government, it is expected that the central government does not want the financial revenue of local government to decrease sharply, so they have the will and courage to discount the implementation of environmental regulations, which is also the main reason why land finance relying on land transfer becomes the source of “second finance”, and this kind of land development mode often brings low density and disordered spread of the city.

Taking Equation (6) into (5), $P=\eta \omega \sqrt{G}-\rho \omega^{2}G$, partial deviation of environmental pollution to population economic density can thus be obtained:

(12) $$\frac{\partial P}{\partial \delta }=\omega \frac{\partial G}{\partial E(\delta )}\frac{\partial E(\delta )}{\delta }(\eta \frac{1}{2\sqrt{G}}-\rho \omega )$$

Because $\frac{\partial G}{\partial E(\delta )}>0$ and $\frac{\partial E(\delta )}{\delta }>0$, hence the sign of $\frac{\partial P}{\partial \delta }$ depends on the value of $\left(\eta \frac{1}{2\sqrt{G}}-\rho \omega \right)$.

When $(\eta \frac{1}{2\sqrt{G}}-\rho \omega )>0$, i.e., $G<\frac{\eta^{2}}{4\rho^{2}\omega^{2}}$, the low density spread of urban population alleviates urban environmental pollution. When $(\eta \frac{1}{2\sqrt{G}}-\rho \omega )<0$, i.e., $G>\frac{\eta^{2}}{4\rho^{2}\omega^{2}}$, the low density spread of urban population exacerbates urban environmental pollution. In other words, there might exist a nonlinear relationship between urban sprawl and environmental pollution.

## 4. Materials and Methods

### 4.1. Variables Selection

(1) Explained variable (POL). Environmental problems are mainly manifested in air, soil, and water pollution. Among them, air pollution has always been an important factor affecting economic and social development as well as people’s lives and health [26]. Due to the difficulties and limitations of data collection, and in order to maintain a certain consistency with other explanatory variables, this paper selected industrial sulfur dioxide emissions per unit area to characterize environmental pollution, which is expressed by POL, and the unit is 10 tons/square kilometer.

(2) Core explanatory variable (SPR). There are several methods to measure urban sprawl, which are mainly divided into the single-index method and multi-index method. Considering the incompleteness of the existing data and the large error of the calculation results, this paper referred to the practice of Song et al. [27] and Yi et al. [28] to characterize urban sprawl by the ratio of the built-up area of the municipal district to the corresponding population.

(3) Control variables.

Investment level (INV). Some scholars [29,30,31] believe that the over development of land market, the prosperity of real estate, and the proposal of real estate tax are the important factors of urban sprawl. At the same time, Bereitschaft and Debbage [32] and Pucher et al. [33] believe that land development, transportation, employment, and other factors affect urban sprawl. Therefore, this paper selected the completion of fixed-assets investment as the control variable.

Industrial structure (STRU). Modern industrial structure has made an important contribution to the growth of urban agglomerations and major cities. Generally speaking, in the process of urbanization, industrial development brings the largest amount of environmental pollution emissions [34]. With the transformation and upgrading of industrial structure, the service industry gets developed vigorously. The higher the proportion of the tertiary industry, the higher the energy utilization efficiency. The development of the tertiary industry facilitates the improvement of energy utilization efficiency. Accelerating the rapid development of the service industry is an important means to optimize the ecological environment of large and medium-sized cities in China. This paper adopted the proportion of added value of the tertiary industry in GDP to characterize the impact of industrial structure on environmental pollution.

Market freedom (FREE). The government plays a leading role in the process of urban development [35,36]. Especially under the performance-oriented assessment mechanism based on GDP, the government tends to be keen on the development of new areas and infrastructure construction, and lacks the necessary regulation of polluting industries, which leads to the inefficient use of land resources and no restrictions on industrial pollution. This paper used the proportion of fiscal revenue to GDP. The larger the ratio, the greater the role of government in resource allocation and the lower the degree of market freedom.

Foreign direct investment (OPEN). FDI is the total amount of foreign capital actually utilized by the city, which is converted into RMB according to the average exchange rate between US dollar and RMB in the corresponding year, and then the proportion in GDP is calculated. There have been controversies about the impact of FDI on environmental pollution, such as “pollution shelter theory” [11] and “pollution halo theory” [37]. Based on the establishment of spatial model, the results showed that the impact of FDI on environmental pollution in different regions and cities with distinct attributes varies. The double effects of “pollution paradise” and “pollution halo” are both verified in China [22].

### 4.2. Data Source

**Figure 3:**
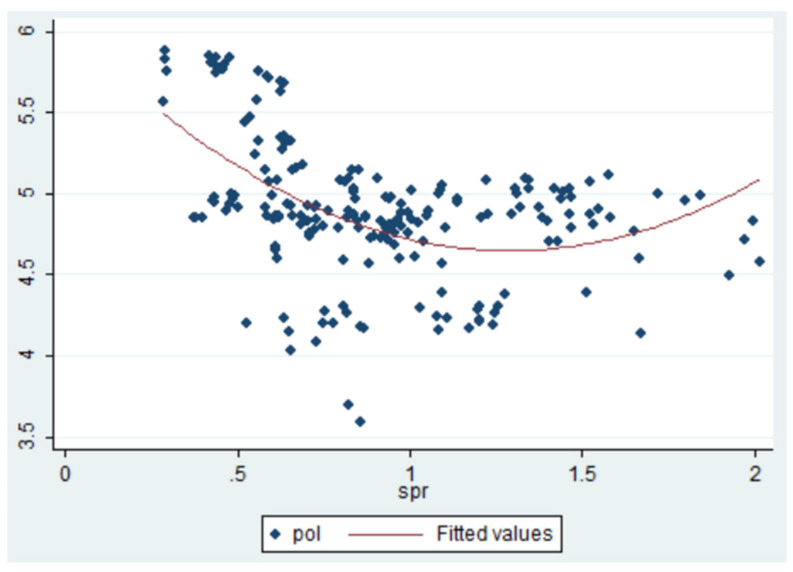
Quadratic fitting analysis of urban sprawl and environmental pollution.

At present, China’s urban sprawl is more serious in large and medium-sized cities [38]. Therefore, this paper chose 35 large and medium-sized cities in China as samples to study the effect of urban sprawl on environmental pollution. Due to the data availability, the sample covers all the sub-provincial cities in China and the provincial capitals of 30 provinces, except Tibet. We not only analyzed the common characteristics of large and medium-sized cities, but also analyzed the regional and intra-regional differences in eastern, central, and Western China so as to obtain rich and substantial research conclusions. In this paper, referring to existing studies and considering the availability of data, the time span of our selected data was 1998–2019, and the above indicators are all from the corresponding annual “China Statistical Yearbook” and “China Urban Statistical Yearbook” (Table 1). The descriptive statistics are shown in Table 2. The mathematical model indicated that urban sprawl impacts environmental pollution in a nonlinear way, in that urban sprawl decreases pollution until a turning point is reached, at which pollution starts to ascend, further visually manifested in the quadratic fitting analysis in Figure 3.

### 4.3. Methods

For panel data, the Ordinary Least Square (OLS) model can be used as a benchmark model. The advantage is simple, but the drawback is that individual effects are not considered. Fixed effect (FE) and random effect (RE) take individual effects into account. When the individual effect is related to the independent variable, the fixed-effect model should be used as the coefficient estimation if the random-effect model is inconsistent. Otherwise, the random effect model is more appropriate. The Hausman test is often used to verify the applicability of the two models.

In the dynamic-panel model setting, the lag term of the explained variable is introduced into the regression model as an explanatory variable, which makes the model have the ability of dynamic interpretation, but there is an endogenous problem in the model. The Systematic Generalized Method of Moments (SYS-GMM) technique estimates the original model and the transformed model with difference, at the same time. The Systematic GMM can correct the unobserved heteroscedasticity problem, missing variable deviation, measurement error, and potential endogenous problems.

In order to prevent spurious regression, the panel-unit root test can be carried out [32]. In this paper, LLC and IPS were used to test the panel data, and the results are shown in Table 3. The test results implied that all of the variables are integrated of the same order, i.e., I (1). This also reveals that all of the variables were stationary at their first-order differentials. The LLC and IPS test both suggested that there might exist a long-run equilibrium relationship among the variables as all the variables are integrated with the same order.

## 5. Results

Before regression, we needed to conduct the VIF (Variance Inflation Factor) test to ensure that there was no multiple linearity between the selected variables [39]. By observing the results in column (6) of Table 4, all the VIF values were between 1.02 and 1.15 (all less than the critical value of 10), which indicates that there was no multicollinearity between them. Secondly, F-statistics showed that the fixed-effect model is better than the OLS-regression model. Next, the Hausman test suggested that the fixed-effect model is better than the random-effect model for columns (4) and (5).

Table 4 reports the estimated results for the full sample. Model (1) regressed without adding control variables, while other models regressed by adding variables step by step. Columns (1) to (6) display that urban sprawl mitigates environmental pollution at the 1% significance level. Since urban sprawl is the expansion of urban low-density, the low-density expansion of the population will subsequently reduce the utilization efficiency of urban public facilities and accelerate the occupation of land resources by repeated construction. However, due to the low density, various activities of residents are relatively scattered and the pressure on the environment is comparatively lower, which is conducive to reducing environmental pollution.

Nevertheless, the regression results of the quadratic term of urban sprawl were significantly positive, which demonstrates that there is a U-shaped curve relationship between urban sprawl and environmental pollution for our selected large and medium-sized cities. This finding is consistent with previous studies [40,41,42,43]. Among other control variables, the fixed-asset investment is significantly positively correlated with environmental pollution; the coefficient of FDI was positive, but not significant in the regression results; the market freedom was significantly negative at the 1% level, indicating that the greater the proportion of fiscal revenue in GDP, the lower the degree of freedom, and thus the more serious the pollution; the coefficient symbol of industrial structure was positive at the 1% significant level.

The coefficient of urban sprawl in each column was negative, and the coefficient of its squared term was positive, and all of them passed the significance test. It shows that there is a U-shaped curve relationship between urban sprawl and environmental pollution, whether for the whole country, small cities, medium-sized cities, large cities, or mega cities. That is, in the initial stage of urban sprawl in China, the expansion of urban space will bring about the decline of environmental pollution. However, when the expansion reaches a certain degree, with the further expansion of urban sprawl, the negative impact of urban sprawl on environmental pollution will be strengthened, and the degree of urban environmental pollution will rise. In other words, at the present stage of development, China’s urban sprawl has not realized the inverted U-shaped Kuznets curve.

### 5.1. Heterogeneity Analysis

(1) Estimation for the three regions

In this paper, 35 large and medium-sized cities were divided into eastern, central, and western regions. There are obvious differences in urban development and environmental conditions in different regions, and the degree of urban sprawl also varies. Therefore, the relationship between urban sprawl and environmental pollution in different regions is likely to be heterogeneous. It is necessary to carry out comparative analysis of the effects of urban sprawl on environmental pollution in different regions. Fixed effect and random effect were also used for estimation. The specific results are shown in Table 5.

Table 5 displays that in general, urban sprawl in the eastern, central, and western regions has a positive impact on environmental pollution, indicating that the impact of urban sprawl on environmental pollution shows strong robustness. Undoubtedly, there are some differences in the impact intensity of urban sprawl on environmental pollution in various regions. The impact of urban sprawl on environmental pollution in the central region was the smallest, followed by the eastern region; the impact intensity of urban sprawl on environmental pollution in the western region was the largest. The fundamental heterogeneity between different regions lies in the level of economic development, and economic capacity does affect the relationship between urban sprawl and environmental pollution. Cities with higher economic development level tended to show higher urbanization rate and a higher degree of urban sprawl. The higher the economic development degree is, the smaller the impact of urban sprawl on environmental pollution. After the incorporation of the quadratic term of urban sprawl index, the relationship between urban sprawl and environmental pollution in the eastern and western regions is U-shaped. With the expansion of urban sprawl, environmental pollution first diminishes and then boosts.

(2) Estimation of cities with different degree of sprawl

There are obvious discrepancies in the extent of urban sprawl, and the impact on environmental pollution may also be significantly different. According to the data characteristics, the low-sprawl type is defined as the sprawl index less than 1, the moderate-sprawl type is defined as the sprawl index between 1 and 1.2, and the high-sprawl type is defined as the sprawl index greater than 1.2. Fixed effect and random effect are also used for estimation. The specific results are shown in Table 6.

Table 6 reports that the effects of cities with a high and low degree of spread on environmental pollution were significantly positive, while those of moderate type were significantly negative. For cities with a distinct degree of urban sprawl, the specific impact of urban sprawl on environmental pollution was different. Cities with a low degree of urban sprawl had the greatest impact on environmental pollution, followed by moderate and high sprawl. After the introduction of the quadratic term of the urban-sprawl index, the relationship between urban sprawl and environmental pollution of high and low sprawl types was U-shaped, while that of moderate sprawl type was inverted U-shaped. In addition, the coefficient of the fixed-asset investment level was significantly positive, the coefficient of opening up was positive but not significant, and the coefficient of market freedom and industrial structure was significantly negative.

(3) Estimation of cities with different pollution levels

The level of pollution in distinct cities was also significantly different, and the impact on environmental pollution may significantly vary. According to the data characteristics, the low-pollution type is defined by a pollution index less than 4, the medium-pollution type is defined by a pollution index between 4.1 and 4.5, and the high-pollution type is defined by a pollution index greater than 4.5. Fixed effect and random effect are also used for estimation. The specific results are shown in Table 7.

Table 7 shows that the effect of urban sprawl on environmental pollution was significantly positive in low pollution cities, significantly negative in medium pollution cities, and not significant in high pollution cities. For cities with different degrees of pollution, the specific effects of urban sprawl on environmental pollution were different. Low pollution cities had the greatest impact on environmental pollution, followed by medium pollution. After incorporating the quadratic term of urban-sprawl index, the relationship between urban sprawl and environmental pollution in low pollution cities was U-shaped, and that between urban sprawl and environmental pollution in medium pollution cities was inverted U-shaped. Additionally, the coefficient of fixed asset investment level was significantly positive, the coefficient of OPEN was negative but not significant, and the coefficient of market freedom and industrial structure had a significant or insignificant negative effect.

### 5.2. Robustness Test

In order to ensure the consistency of the estimation results, we needed to perform a series of robustness tests on the analysis results. Due to the long time span of the selected samples, in order to eliminate the impact of time trend on the empirical results [37], this paper divided the whole sample into two periods: 1998–2008 and 2009–2019. The analysis results are shown in columns (1), (2), (3), and (4) of Table 8. The coefficient of the core explanatory variable was still significant, and there was temporal heterogeneity. From 1998 to 2008, urban sprawl had an inverted U-shaped impact on environmental pollution, while in 2009–2019, urban sprawl had a U-shaped impact on environmental pollution.

The existence of endogenous variables in the model will have a greater impact on the estimation results of the model, and the change of urban sprawl will also have an impact on environmental pollution. In addition, changes in environmental pollution may in turn affect urban sprawl. In order to eradicate the influence of endogenous variables, a dynamic panel model was constructed, and the systematic generalized-moment estimation method was used to calculate the model. The estimation results are displayed in Table 8. AR (1) and AR (2) showed that there is first-order autocorrelation in the difference of disturbance term, but there was no second-order autocorrelation. Therefore, systematic GMM estimation can be utilized. At the same time, the Sargan test also suggested that all instrumental variables were valid and the model specification was reasonable. The estimated results in Table 8 imply that urban sprawl and environmental pollution have a U-shaped feature, which is consistent with the previous results. The coefficient sign and significance of other control variables were basically consistent with the previous, indicating that the regression results were robust.

### 5.3. Discussion

Cities including Huhhot, Hefei, Xiamen, and Qingdao have low-density expansion of urban space; however, the extent of urban sprawl in Shanghai, Harbin, Chongqing, Xi’an, and Hangzhou is not obvious. Cities with serious environmental pollution problems contain Tianjin, Shijiazhuang, Fuzhou, Guiyang, and the like; environmental pollution in Beijing, Taiyuan, Changsha, and other cities is constantly improving (Table 9).

The research results were basically consistent with those of scholars from studying other countries such as the United States and Europe [44,45]. Urbanization is an important reason to promote the increase of total carbon emissions in the United States [46]. Navamuel et al. [2] discussed the impact of urban sprawl on energy consumption and carbon emissions in Spain. The results showed that urban sprawl makes people more dependent on cars, resulting in a significant increase in energy consumption and carbon emissions in the region.

In addition, our study also found an interesting phenomenon: the impact intensity of urban sprawl on ecological environment gradually weakens with the increase of sprawl degree. This is similar to the research of Qin and Liu [38], who found that the impact of urban sprawl on productivity in China is gradually decreasing. With the advancement of urbanization, the impact of urban sprawl on productivity and energy efficiency is weakening. The reason would be that a larger urban scale can promote infrastructure construction, improve the level of division of labor and production technology, and uses resources more efficiently so as to reduce the energy consumption per unit output. The policies and plans related to urban spatial structure are adjusted correspondingly in the process of regional development, and thus the impact on the environment is gradually reduced.

## 6. Conclusions and Policy Implications

This paper selected panel data from 1998 to 2019 across 35 large and medium-sized cities in China to empirically analyze the effect of urban sprawl on environmental pollution. The research findings showed that urban sprawl has a nonlinear correlation with the ecological environment. Furthermore, the regression results demonstrated a U-shaped curve relationship between urban sprawl and environmental degradation. That is to say, urban expansion benefits emission reduction and pollution governance for the early stage; nevertheless, the environmental condition will be worsened along with the further spread of the urban boundary. Based upon the research conclusions, the policy recommendations are put forward as follows.

(1) Scientifically planning urban construction, and reasonably controlling urban sprawl. The overall level of urban sprawl in the municipal cities is relatively high, and the urban sprawl index is increasing year by year. The obvious feature of urban sprawl is that land urbanization develops faster than population urbanization, and the current situation of urban sprawl in large and medium-sized cities leads to the aggravation of environmental pollution. Therefore, the government should reasonably plan urban construction according to the theories of smart growth, intensive land use, and the urban growth boundary, and reasonably regulate and control the development process of land urbanization and population urbanization. Moderate control of the growth trend of urban sprawl is the key to achieve the coordinated development of urbanization and environmental governance.

(2) The scientific combination of new urbanization and ecological civilization construction, integration of ecological civilization construction into the whole process of new urbanization development, and realizing the interactive development of the two have important practical guiding significance for China’s new urbanization construction, ecological civilization construction, and even the sustainable development of national economy. The concept of green, low-carbon, and ecological development should be integrated into the whole process of new urbanization. At the same time, it is also necessary to formulate corresponding policies to reduce its negative impact. By means of carbon tax, emission trading, and energy price marketization, we can adjust the energy consumption structure and improve the energy efficiency so as to solve the environmental pollution problems in the process of urbanization in China.

(3) Discreetly determining urban boundaries. Urban sprawl has a direct and indirect impact on environmental pollution, which is one of the most significant reasons for urban environmental pollution. Therefore, the government should strengthen governance to avoid urban sprawl. In order to control urban sprawl and protect the ecological environment, the Ministry of Housing and Urban Rural Development and the Ministry of Land and Resources identified 14 pilot cities, including Beijing and Shanghai, in July 2014 to explore the delineation of urban development boundaries. In 2018, the Ministry of Land and Resources issued the administrative measures for overall land use planning, which stipulates the preparation of overall land use planning at the municipal, county, and township levels, and requires the demarcation of urban and rural construction land-scale boundaries, expansion boundaries, and forbidden construction boundaries according to local conditions. At present, it is necessary to speed up the process of delimiting urban boundaries and reasonably determine the boundaries of each city according to scientific principles so as to avoid the spread of low density.

(4) Eastern and mega-cities should actively guide residents to change their consumption concepts, enhance their awareness of environmental protection, and reduce domestic pollution. These cities should take the lead in issuing corresponding laws and regulations, strengthen the publicity and education of environmental protection knowledge, strengthen the awareness of environmental protection and resource conservation of citizens, and improve the public participation in environmental protection so as to effectively reduce the residents’ domestic sewage and build a beautiful city. Central/western and big cities should speed up the transformation of the economic development mode and try their best to reduce industrial pollution. They should actively transform the mode of economic growth, enhance the ability of scientific and technological innovation, promote industrial transformation and upgrading, and gradually reduce industrial pollution.

## Figures and Tables

**Table 1:** City names and major socioeconomic conditions in 2019.

City	Population (Million)	Growth Rate of Population 1998–2019 (%)	Growth Rate of Built-Up Area 1998–2019 (%)	Added Value of Tertiary Industry to GDP (%)	Education Expenditure to GDP (%)	Per Capita Income ($)
Beijing	13.97	42.28	201.02	83.52	3.21	23,805.18
Tianjin	11.08	85.57	210.24	63.46	3.31	13,100.09
Shijiazhuang	4.27	171.68	194.29	70.54	3.24	10,212.08
Taiyuan	3	34.34	92.09	63.19	1.01	14,840.62
Huhhot	1.41	38.55	148.57	73.22	1.13	10,223.09
Shenyang	6.13	27.71	178.71	63.14	1.30	12,416.61
Dalian	4.05	54.34	89.74	56.73	1.57	14,481.41
Changchun	4.45	57.42	274.48	52.21	1.74	17,547.87
Harbin	5.53	84.85	102.73	70.58	2.01	10,748.57
Shanghai	14.69	37.21	125.09	72.73	2.61	22,799.01
Nanjing	7.1	156.91	359.78	62.02	2.06	24,016.96
Hangzhou	6.57	283.22	282.43	67.51	2.32	23,517.58
Ningbo	3.01	151.48	454.69	54.8	2.09	25,006.60
Hefei	2.91	127.45	300.83	64.65	1.88	23,420.31
Fuzhou	2.9	101.82	276.25	63.54	1.67	20,918.17
Xiamen	2.61	106.18	503.03	57.96	2.46	20,691.31
Nanchang	3.14	93.70	336.76	52	1.29	16,494.31
Jinan	6.95	130.42	522.61	61.74	2.22	21,155.90
Qingdao	5.29	169.91	564.91	63.75	1.53	15,585.27
Zhengzhou	3.97	94.67	400.86	63.47	2.06	19,138.94
Wuhan	9.06	70.87	298.04	60.75	1.79	21,098.06
Changsha	3.64	118.08	320.87	67.12	1.93	23,839.24
Guangzhou	9.54	138.92	381.45	71.62	2.22	22,675.51
Shenzhen	5.51	380.80	619.38	60.93	2.66	29,497.57
Nanning	3.98	204.26	251.65	70.76	2.63	11,936.51
Haikou	1.83	246.66	476.47	79.23	2.48	10,468.65
Chongqing	24.79	196.33	533.89	53.81	2.19	12,130.61
Chengdu	8.76	168.73	394.79	70.28	1.31	17,825.90
Guiyang	2.67	51.20	276.53	62.34	2.88	13,392.33
Kunming	3.25	87.80	237.88	68.57	1.64	16,675.07
Xi’an	8.21	119.08	332.72	63.18	1.37	14,536.20
Lanzhou	2.12	21.16	44.17	68.38	2.80	12,280.93
Xining	1.01	22.77	63.33	76.07	1.50	10,600.71
Yinchuan	1.25	113.31	297.92	69.49	1.40	11,246.07
Urumqi	2.22	59.49	248.57	72.82	2.59	9,355.51

**Table 2:** Descriptive statistics of the panel data.

Variable	Mean	Std.Dev.	Min	Max
POL	4.7782	0.5303	1.9637	5.8801
SPR	0.9412	0.4528	0.2856	3.9191
SPR^2^	1.0906	1.5129	0.0816	15.3592
INV	0.5421	0.2256	0.1231	1.4533
OPEN	0.0472	0.0403	0.0001	0.3839
FREE	0.0941	0.0345	0.0071	0.2273
STRU	0.5451	0.0836	0.3795	0.8056

**Table 3:** Panel-unit root test results.

Levin-Lin-Chu (LLC) Test				
	Level		First Difference	
	Intercept	Intercept and Trend	Intercept	Intercept and Trend
POL	0.3335	−1.4773 **	−14.0974 ***	−4.5209 ***
SPR	−1.1902 *	−3.6850 ***	−8.2394 ***	−6.9605 ***
**Im-Pesaran-Shin (IPS) Test**				
	Level		First difference	
	Intercept	Intercept and trend	Intercept	Intercept and trend
POL	1.4760	−0.7613	−11.4293 ***	−6.4110 ***
SPR	−0.2364	−0.0010	−8.2513 ***	−6.5611 ***

Note: * *p* < 0.1. ** *p* < 0.05. *** *p* < 0.01.

**Table 4:** Estimation results of the full sample.

Independent Variable	(1) ln *POL*	(2) ln *POL*	(3) ln *POL*	(4) ln *POL*	(5) ln *POL*	(6) VIF Test
SPR	−0.5815 *** (−7.07)	−0.7618 *** (−8.16)	−0.7925 *** (−8.02)	−0.6639 *** (−3.23)	−0.3910 *** (−3.57)	1.10
*(SPR)^2^*	0.1439 *** (5.61)	0.1852 *** (6.75)	0.1897 *** (6.83)	0.1571 *** (5.35)	0.0994 *** (3.36)	
INV		0.2418 *** (3.93)	0.2435 *** (3.87)	0.1994 *** (3.15)	0.1851 *** (3.07)	1.02
OPEN			0.2729 (0.76)	0.6321 ** (1.75)	0.3069 (0.89)	1.12
FREE				−2.8035 *** (−5.51)	−1.8094 *** (−3.62)	1.15
STRU					−1.7743 *** (−8.04)	1.13
_cons	4.9328 *** (58.18)	4.9073 *** (56.87)	4.8689 *** (53.65)	5.0443 *** (−4.06)	5.8483 *** (51.30)	
R squared	0.0408	0.0313	0.0176	0.0921	0.1780	
F-statistic or Wald	20.82 [0.00]	22.85 [0.00]	31.08 [0.00]	15.70 [0.00]	26.76 [0.00]	
Hausman Test	0.20 [0.91]	1.77 [0.62]	4.39 [0.36]	8.15 [0.14]	17.24 [0.00]	
Model	RE	RE	RE	FE	FE	

Note: the value in the parenthesis is t-statistic or z-statistic. ** *p* < 0.05. *** *p* < 0.01.

**Table 5:** Estimation results of subgroups categorized by region.

Independent Variable	(1) Eastern	(2) Eastern	(3) Central	(4) Central	(5) Western	(6) Western
SPR	−0.9141 *** (−7.77)	−0.6924 *** (−5.02)	0.1475 (0.45)	−0.1713 (−0.49)	−1.2037 ** (−3.97)	−1.8071 *** (−5.11)
(SPR)^2^	0.2102 *** (6.75)	0.1541 *** (4.36)	−0.1581 (−0.92)	−0.0952 *** (−0.56)	0.4696 *** (3.65)	0.7109 *** (4.61)
INV		0.7023 *** (4.60)		0.2097 ** (2.12)		
OPEN				0.0444 (0.09)		0.0522 (−0.04)
FREE		−4.1582 *** (−8.65)		1.4551 * (1.52)		−2.9757 *** (−3.04)
STRU				−0.6468 (−1.38)		
_cons	5.0971 *** [92.23]	5.4336 *** [65.58]	4.6779 *** (24.32)	4.9515 *** (19.67)	5.0016 *** (41.22)	5.3129 *** (33.16)
R squared	0.5422	0.2809	0.0626	0.0733	0.2668	0.1078
F-statistic or Wald	13.37 [0.00]	31.64 [0.00]	3.68 [0.05]	3.48 [0.00]	2.87 [0.09]	18.50 [0.00]
Hausman Test	3.84 [0.14]	10.14 [0.03]	0.92 [0.63]	32.42 [0.00]	1.83 [0.40]	2.42 [0.65]
Model	FE	FE	RE	FE	RE	RE

Note: the values in the parentheses are t-statistic or z-statistic. * *p* < 0.1. ** *p* < 0.05. *** *p* < 0.01.

**Table 6:** Estimation results of subgroups categorized by urban sprawl.

Independent Variable	(1) High Sprawl	(2) High Sprawl	(3) Middle Sprawl	(4) Middle Sprawl	(5) Low Sprawl	(6) Low Sprawl
SPR	−0.7562 *** (−6.19)	−0.7341 *** (−5.14)	0.7188 * (1.44)	0.8056 ** (1.69)	−1.8369 *** (−2.41)	−2.0484 *** (−2.60)
(SPR)^2^	0.1866 *** (5.61)	0.1777 *** (4.70)	−0.5510 ** (−1.93)	−0.4564 ** (−1.69)	1.0132 ** (1.90)	1.1337 ** (2.06)
INV		0.1504 (1.20)				0.1717 ** (1.82)
OPEN				0.0061 (0.01)		0.3324 (0.35)
FREE		−1.5518 * (−1.57)		−0.5084 (−0.61)		−1.5307 * (−1.59)
STRU				−2.2162 *** (−5.64)		
_cons	4.8758 *** [54.32]	5.1359 *** [42.20]	4.9187 *** (73.76)	5.8003 *** (29.52)	5.2238 *** (39.72)	5.3032 *** (31.62)
R squared	0.3161	0.1938	0.2922	0.2222	0.3705	0.0864
F-statistic or Wald	9.10 [0.00]	20.18 [0.00]	9.65 [0.00]	13.09 [0.00]	10.27 [0.00]	16.89 [0.00]
Hausman Test	1.77 [0.41]	3.18 [0.52]	5.20 [0.07]	21.34 [0.00]	2.82 [0.24]	3.34 [0.64]
Model	RE	FE	FE	FE	RE	RE

Note: the values in the parentheses are t-statistic or z-statistic. * *p* < 0.1. ** *p* < 0.05. *** *p* < 0.01.

**Table 7:** Estimation results of subgroups categorized by environmental pollution.

Independent Variable	(1) High Pollution	(2) High Pollution	(3) Middle Pollution	(4) Middle Pollution	(5) Low Pollution	(6) Low Pollution
SPR	−0.2662 (−0.99)	0.0333 (1.60)	0.0749 (0.25)	0.8091 *** (2.44)	−0.1931 *** (−2.92)	−1.6936 *** (−6.88)
(SPR)^2^	0.1178 (1.03)	0.0428 (4.70)	−0.1602 (−0.95)	−0.5162 *** (−2.90)	1.0132 ** (1.90)	0.3779 *** (6.46)
INV		−0.0724 (−0.98)		0.2252 *** (2.69)		0.5121 *** (3.75)
OPEN				−0.3714 (−0.63)		−0.7263 (−1.19)
FREE		−1.7344 ** (−2.35)		−0.8133 (−1.35)		0.2706 (0.20)
STRU		−0.4003 (−1.21)		−2.2717 *** (−7.25)		
_cons	5.0033 *** [80.91]	5.3006 *** [34.28]	5.0390 *** (62.05)	6.1729 *** (38.35)	4.6416 *** (20.28)	4.9117 *** (33.33)
R squared	0.4030	0.1863	0.0436	0.3142	0.0443	0.1195
F-statistic or Wald	0.00 [0.97]	2.80 [0.02]	13.23 [0.00]	23.18 [0.00]	8.52 [0.00]	6.31 [0.00]
Hausman Test	5.09 [0.07]	8.67 [0.12]	0.00 [0.99]	31.14 [0.00]	0.28 [0.86]	10.75 [0.05]
Model	FE	FE	RE	FE	RE	FE

Note: the value-s in the parentheses are t-statistic or z-statistic. ** *p* < 0.05. *** *p* < 0.01.

**Table 8:** Robustness check.

Independent Variable	(1) 1998–2008	(2) 1998–2008	(3) 2009–2019	(4) 2009–2019	(5) SYS-GMM	(6) SYS-GMM
L1.POL					1.1344 *** (128.74)	1.0675 *** (69.35)
SPR	0.2552 *** (2.76)	0.2949 *** (2.58)	−1.1658 *** (−5.44)	−1.3040 *** (−5.62)	−0.3769 *** (−34.51)	−0.0756 *** (−3.28)
(SPR)^2^	−0.0442 ** (−1.98)	−0.0512 ** (−1.90)	0.3047 *** (4.66)	0.3450 *** (2.58)	0.0879 *** (38.13)	0.0160 *** (3.76)
INV		0.0504 (0.69)		0.2369 ** (1.77)		0.0576 *** (2.58)
OPEN		0.7315 *** (2.56)		−0.1165 (−0.11)		
FREE		−0.6564 ** (−1.18)		−1.2629 (−1.37)		−0.6950 *** (−3.71)
STRU		0.3971 * (1.46)				−1.0816 *** (−18.21)
_cons	4.7827 *** (52.17)	4.5548 *** (33.19)	4.9915 *** (40.64)	4.9507 *** (29.88)	−0.5148 *** (−12.58)	0.3228 *** (3.31)
R squared	0.3515	0.3515	0.0654	0.0423		
F-statistic or Wald	3.97 [0.00]	3.97 [0.00]	8.13 [0.00]	10.71 [0.03]		
Hausman Test	1.19 [0.55]	30.47 [0.00]	0.02 [0.99]	0.00 [0.99]		
AR(1)					0.0034	0.0031
AR(2)					0.9784	0.9841
Sargan test					34.6741 [0.9691]	33.3532 [0.9794]
Model	RE	FE	RE	RE	SYS-GMM	SYS-GMM

Note: the values in the parentheses are t-statistic or z-statistic. * *p* < 0.1. ** *p* < 0.05. *** *p* < 0.01.

**Table 9:** Detailed classification and impact characteristics.

	(1) High Sprawl	(2) Middle Sprawl	(3) Low Sprawl	(4) High Pollution	(5) Middle Pollution	(6) Low Pollution
Cities	Huhhot Hefei Xiamen Qingdao Zhengzhou Changsha Guangzhou Shenzhen Guiyang Kunming Yinchuan Urumqi	Beijing Tianjin Taiyuan Dalian Changchun Nanjing Ningbo Fuzhou Nanchang Wuhan Haikou Chengdu Lanzhou	Shijiazhuang Shenyang Harbin Shanghai Hangzhou Jinan Nanning Chongqing Xi’an Xining	Tianjin Shijiazhuang Huhhot Dalian Fuzhou Chongqing Guiyang Kunming Xining Urumqi	Shenyang Changchun Harbin Shanghai Nanjing Hangzhou Ningbo Nanchang Jinan Zhengzhou Wuhan Guangzhou Chengdu Lanzhou Yinchuan	Beijing Taiyuan Hefei Xiamen Qingdao Changsha Shenzhen Nanning Haikou Xi’an
Impact on pollution	significant U-shaped curve relationship	significant inverted U-shaped curve relationship	significant U-shaped curve relationship	insignificant	significant inverted U-shaped curve relationship	significant U-shaped curve relationship

## Data Availability

China Statistics Yearbook 1999–2020. http://www.stats.gov.cn/tjsj/ndsj/ (accessed on 8 August 2021).
